# Supplementary material for: Annual phytoplankton dynamics in coastal waters from Fildes Bay, Western Antarctic Peninsula
Source: Sci Rep. 2021 Jan 14;11:1368. doi: 10.1038/s41598-020-80568-8 (PMC7809266; doi:10.1038/s41598-020-80568-8)
Supplement: Supplementary file 1 — Supplementary Information. [file 41598_2020_80568_MOESM1_ESM.pdf]

Annual phytoplankton dynamics in coastal waters from Fildes Bay, Western Antarctic Peninsula.

Nicole Trefault[1+*], Rodrigo De la Iglesia[2+], Mario Moreno-Pino[1], Adriana Lopes dos Santos[3], Catherine Gérikas Ribeiro[1], Génesis Parada-Pozo[1], Antonia Cristi[1], Dominique Marie[4], and Daniel Vaulot[4, 3*]

[1]GEMA Center for Genomics, Ecology & Environment, Faculty of Sciences, Universidad Mayor, Santiago, 8580745, Chile

[2]Department of Molecular Genetics and Microbiology, Pontificia Universidad Católica de Chile, Alameda 340, Santiago, 8331150, Chile

[3]Asian School of the Environment, Nanyang Technological University, 50 Nanyang Avenue, Singapore 639798

[4]Sorbonne Université, CNRS, UMR7144, Ecology of Marine Plankton team, Station Biologique de Roscoff, 29680 Roscoff, France

[*]Corresponding authors: nicole.trefault@umayor.cl, vaulot@gmail.com

[+]These authors contributed equally to this work

Supplementary material

Published in **Scientific Reports**
December 14, 2020

Supplementary Data

All supplementary material is available at https://github.com/vaulot/Paper-Trefault-2020-Antarctica

- **Supplementary Data S1**: List of metabarcoding samples with environmental data (Antarctica_2015_samples.xlsx).
- **Supplementary Data S2**: List of classes, genera and species found by each metabarcoding approach in surface samples from summer 2015. Taxa with uncertain affiliation (labelled by _X in the PR2 database) were not taken into account (dada2/method_comparison.xlsx).
- **Supplementary Data S3**: List of ASVs for 18S rRNA gene of filtered samples with abundance table for the different samples - see for sample codes in Supplementary Data S1 (dada2/metapr2_wide_asv_set_16_photo.xlsx).
- **Supplementary Data S4**: List of ASVs for plastidial 16S rRNA gene of filtered samples with abundance table for the different samples - see for sample codes in Supplementary Data S1 (dada2/metapr2_wide_asv_set_17_photo.xlsx).
- **Supplementary Data S5**: List of ASVs for plastidial 16S rRNA gene of filtered samples with abundance table for the different samples - see for sample codes in Supplementary Data S1 (dada2/metapr2_wide_asv_set_18_photo.xlsx).
- **Supplementary Data S6**: Script used to process the data with output (R markdown): https://vaulot.github.io/Paper-Trefault-2020-Antarctica/Antarctica-phyloseq.html.

Table S1. Metadata available for the vertical profile samples of January 16, 2015. PPE, PNE, CRY corresponds to abundance of photosynthetic pico-eukaryotes, nano-eukaryotes and cryptophytes, respectively, in cell mL^{-1}.

Depth (m)	T (°C)	PSU	Chl-a	NO_3^-	NO_2^-	PO_4^{3-}	${SiO_3}^{2-}$	PPE	PNE	CRY
5	1.12	33.9	2.63	12.0	0.28	1.08	34.5	3301	10138	788
15	1.05	34.0	1.75	7.8	0.20	1.04	20.4	2605	6366	519
20	1.05	34.0	1.56	11.2	0.25	1.20	33.2	1960	6076	281
25	1.02	34.0	1.73	10.4	0.20	1.20	32.0	2115	5663	227
50	0.80	34.0	1.46	13.6	0.25	1.35	34.4	2130	5062	336

Table S2. List of species in the metabarcoding data sets only found in the deep samples (from 10 to 50 m).

Division	Class	Species
Chlorophyta	Chlorophyceae	*Chlamydomonas acidophila*
		Coccomyxa sp.
		Haematococcus zimbabwiensis
		Hydrodictyon reticulatum
		Oophila amblystomatis
		Planophila sp.
		Radiococcus polycoccus
	Trebouxiophyceae	*Chlorella mirabilis*
		Chlorella sorokiniana
		Chlorella sp.
		Chlorella vulgaris
		Desmococcus endolithicus
		Koliella sempervirens
		Stichococcus bacillaris
		Trebouxia sp.
Cryptophyta	Cryptophyceae	*Chroomonas* sp.
		Hemiselmis sp.
		Teleaulax sp.
Haptophyta	Prymnesiophyceae	*Phaeocystis pouchetii*
Ochrophyta	Bacillariophyta	*Amphora* sp.
		Bacillaria paxillifer
		Coscinodiscus jonesianus
		Gyrosigma limosum
		Navicula lanceolata
		Nitzschia dissipata
		Nitzschia sp.
		Pauliella toeniata
		Pinnularia microstauron
		Proboscia inermis
		Pseudo-nitzschia turgidula

Division	Class	Species
		Rhizosolenia imbricata var shrubsolei
		Rhizosolenia setigera
		Synedropsis recta
		Tabularia sp.
		Tabularia tabulata
		Thalassionema nitzschioides
		Thalassiosira nordenskioeldii
		Ulnaria acus
	Bolidophyceae	*Triparma mediterranea*
	Dictyochophyceae	*Mesopedinella arctica*
		Pseudochattonella verruculosa
		Pteridomonas danica

Table S3. List of species found in the metabarcoding data sets for the surface samples. Minimum (min), mean (mean) and Maximum (max) contribution (in %) to the photosynthetic metabarcodes and the number of samples (n) where found for the 18S-filter, 16S-filter and 18S-sort datasets.

Division	Class	Species	18S rRNA filter				16S rRNA plastid filter				18S rRNA sort			
			min	mean	max	n	min	mean	max	n	min	mean	max	n
Chlorophyta	Chlorophyceae	*Chlamydomonas hedleyi*	0.03	0.04	0.04	2								
		Chlamydomonas kuwadae	0.02	0.04	0.09	4								
		Chlamydomonas raudensis	0.01	0.01	0.01	1								
		Pleurastrum sp.	0.04	0.04	0.04	1								
	Mamiellophyceae	*Bathycoccus prasinos*	0.02	7.83	66.17	38	0.01	0.21	0.83	10	1.08	2.40	4.32	8
		Mantoniella squamata	0.07	0.08	0.09	3	0.03	0.31	1.33	12				
		Micromonas clade B3	0.04	3.05	12.27	14					0.14	2.08	4.04	7
		Micromonas polaris	0.01	5.08	46.68	42					0.06	14.70	41.10	14
	Palmophyllophyceae	*Prasinoderma coloniale*					0.01	0.11	0.54	17				
		Prasinoderma sp.	0.01	0.13	0.42	19					0.16	0.16	0.16	1
	Pyramimonadophyceae	*Pyramimonas australis*	0.01	0.52	4.21	33					0.02	0.38	0.99	3
		Pyramimonas disomata					0.00	0.01	0.02	3				
		Pyramimonas gelidicola	0.01	1.28	7.82	44					0.92	5.68	10.20	8
		Pyramimonas sp.					0.14	11.31	36.88	40				
	Trebouxiophyceae	*Chloroidium ellipsoideum*	0.02	0.02	0.02	1								
		Chloroidium saccharophila	0.04	0.04	0.04	1								
		Prasiola crispa	0.01	0.04	0.09	3								
	Ulvophyceae	*Acrochaete leptochaete*	0.00	0.06	0.24	15								
		Acrosiphonia sp.					0.05	0.05	0.05	1				
		Chlorothrix sp.	0.04	0.20	0.61	16								
		Dilabifilum sp.	0.01	0.11	0.19	6								
		Monostroma grevillei	0.03	0.16	0.56	13								
		Ulothrix zonata	0.17	0.17	0.17	1								
Cryptophyta	Cryptophyceae	*Falcomonas daucoides*									0.05	0.08	0.12	2
		Falcomonas sp.	0.01	0.01	0.01	2								
		Geminigera cryophila	0.04	13.05	56.48	50	0.01	0.01	0.01	1	1.38	15.62	24.37	10
		Hemiselmis tepida	0.11	0.32	0.51	4								
		Plagioselmis nannoplanctica	0.05	0.18	0.31	2								
Haptophyta	Prymnesiophyceae	*Chrysochromulina* sp.	0.02	0.15	0.43	25	0.11	22.96	80.47	39	0.65	1.61	2.56	8
		Dicrateria sp.					0.01	0.04	0.08	12				
		Phaeocystis antarctica	0.03	2.04	8.22	47					4.04	18.38	31.98	15
		Phaeocystis cordata					0.01	0.01	0.01	1				
		Phaeocystis sp.	0.03	0.04	0.05	2	0.07	10.94	48.49	39	0.68	1.53	2.53	3
		Prymnesium parvum					0.02	0.02	0.02	1				
		Prymnesium pigrum					0.01	0.01	0.02	2				
Ochrophyta	Bacillariophyta	*Achnanthes bongranii*	0.01	0.01	0.01	2								

Division	Class	Species	18S filter				16S filter				18S sort			
			min	mean	max	n	min	mean	max	n	min	mean	max	n
		Actinocyclus actinochilus	0.01	0.06	0.23	16								
		Actinocyclus curvatulus	0.02	0.06	0.10	5								
		Amphora proteus	0.02	0.11	0.54	11								
		Asteromphalus sp.	0.02	0.03	0.05	2								
		Asteroplanus karianus	0.02	0.44	2.75	46	0.01	0.03	0.05	2	0.25	0.87	1.95	8
		Chaetoceros danicus	0.05	0.09	0.14	5								
		Chaetoceros debilis 2	0.02	0.24	0.76	17								
		Chaetoceros dichaeta	0.04	0.14	0.31	6								
		Chaetoceros gelidus	0.06	0.08	0.11	3								
		Chaetoceros neogracilis	0.05	0.68	1.83	46					0.06	13.43	35.09	12
		Chaetoceros peruvianus	0.00	0.01	0.02	3								
		Chaetoceros rostratus	0.04	0.11	0.19	6								
		Chaetoceros socialis	0.02	0.45	2.42	26					0.02	0.56	0.98	4
		Chaetoceros sp.					0.08	0.59	1.81	25				
		Cocconeis stauroneiformis	0.55	0.55	0.55	1								
		Conticribra weissflogii					0.01	0.01	0.01	1				
		Corethron inerme	0.02	1.37	8.39	50								
		Corethron pennatum					0.02	3.25	53.08	28				
		Coscinodiscus concinnus	0.03	0.03	0.03	1								
		Coscinodiscus sp.					0.11	0.11	0.11	1				
		Cyclotella sp.					0.05	0.05	0.05	1				
		Cylindrotheca closterium					0.03	0.03	0.03	1				
		Cymatosira belgica					0.04	0.04	0.04	1				
		Cymbella gastroides	0.01	0.11	0.50	14								
		Cymbella laevis	0.02	0.03	0.04	2								
		Cymbella salina	0.04	0.04	0.04	1								
		Dickieia ulvacea	0.02	0.02	0.02	1								
		Ditylum brightwellii					0.02	0.02	0.02	1				
		Ditylum sol	0.10	0.11	0.13	2								
		Encyonema sp.	0.01	0.09	0.28	13								
		Eucampia antarctica	0.02	0.19	0.68	18								
		Eucampia zodiacus					0.02	0.05	0.07	2				
		Fragilariopsis cylindrus	0.29	5.69	25.03	50	0.04	1.70	10.38	36	0.12	12.15	26.26	16
		Fragilariopsis sublineata	0.11	0.28	0.53	17					0.54	0.62	0.77	3
		Grammonema striatula	0.01	0.05	0.16	8								
		Grammonema striatulum					0.11	0.25	0.47	3				
		Guinardia delicatula	0.02	0.03	0.04	3								
		Guinardia solstherfothii	0.07	0.07	0.07	2								
		Haslea spicula	0.01	0.12	0.34	19								
		Hemiaulus sinensis	0.03	0.10	0.19	7								

Division	Class	Species	18S filter				16S filter				18S sort			
			min	mean	max	n	min	mean	max	n	min	mean	max	n
		Lauderia annulata					0.01	0.01	0.01	1				
		Licmophora grandis	0.06	0.19	0.53	7								
		Minidiscus sp.	0.67	18.54	58.94	49	0.39	13.87	53.84	38	1.78	19.62	31.99	10
		Minidiscus trioculatus	0.18	1.54	2.83	5								
		Navicula perminuta	0.01	0.01	0.01	1								
		Navicula phyllepta					0.28	0.29	0.30	2				
		Navicula sp.	0.04	0.16	0.55	9								
		Odontella aurita	0.01	0.01	0.01	1								
		Odontella mobiliensis	0.06	0.07	0.08	2								
		Odontella sinensis					0.08	0.62	1.80	24				
		Phaeodactylum tricornutum					0.13	0.13	0.13	1				
		Pleurosigma intermedium	0.01	0.01	0.01	1								
		Podosira stelligera					0.03	0.03	0.03	1				
		Porosira glacialis	0.09	18.41	73.84	49	0.19	17.25	85.02	36	0.43	1.68	2.94	2
		Porosira pseudodelicatula	0.01	0.03	0.04	2								
		Porosira pseudodenticulata	0.01	0.01	0.02	2								
		Porosira sp.	0.01	0.01	0.01	1								
		Proboscia alata	0.02	0.24	0.92	22	0.02	0.47	1.95	28				
		Proboscia sp.					0.03	0.03	0.03	1				
		Pseudo-nitzschia seriata	0.03	0.82	4.80	36					0.06	0.40	0.88	3
		Pseudo-nitzschia sp.					0.07	1.23	11.39	20	0.20	0.77	1.33	2
		Pseudogomphonema sp.	0.02	0.09	0.28	7								
		Pteroncola inane	0.01	0.03	0.05	5								
		Rhizosolenia fallax	0.02	0.02	0.02	1								
		Shionodiscus ritscheri	0.05	0.38	1.02	12								
		Skeletonema costatum					0.01	0.01	0.01	1				
		Skeletonema sp.									0.00	0.00	0.00	1
		Stellarima microtrias	0.01	0.03	0.08	8	0.46	0.46	0.46	1				
		Synedra hyperborea	0.03	0.03	0.03	1								
		Synedropsis hyperborea					0.04	0.04	0.04	1				
		Thalassionema frauenfeldii	0.07	0.07	0.07	2	0.02	0.11	0.25	4				
		Thalassiosira antarctica	0.37	7.30	26.50	29								
		Thalassiosira minima	0.18	3.96	11.46	49					0.12	3.12	9.40	13
		Thalassiosira oceanica	0.04	0.04	0.04	1								
		Thalassiosira oestrupii	0.02	0.02	0.02	1								
		Thalassiosira rotula	0.10	0.10	0.10	1								
		Thalassiosira sp.	0.15	3.11	16.04	38	0.18	4.78	19.85	19	0.28	5.99	15.03	5
		Thalassiosira tumida	0.02	0.26	0.87	9								
		Thalassiothrix longissima	0.01	0.08	0.21	7								
	Bolidophyceae	*Triparma laevis clade*	0.03	0.56	2.70	49					0.05	2.01	7.85	10

Division	Class	Species	18S filter				16S filter				18S sort			
			min	mean	max	n	min	mean	max	n	min	mean	max	n
		Triparma laevis f. inornata					0.06	0.69	2.36	37				
		Triparma pacifica					0.04	0.12	0.34	5				
		Triparma sp.	0.04	0.34	0.61	4								
	Dictyochophyceae	*Dictyocha speculum*	0.05	0.11	0.25	4								
		Florenciella parvula	0.02	0.48	1.90	36	0.02	0.13	0.35	6	0.07	0.09	0.11	2
		Helicopedinella sp.					0.01	0.38	1.74	23				
		Pseudochattonella farcimen	0.01	0.30	1.01	32					0.27	0.52	0.77	2
		Pseudochattonella sp.	0.01	0.07	0.15	12					0.14	0.14	0.14	1
		Pseudopedinella sp.	0.01	0.01	0.01	1								
	Pelagophyceae	*Pelagomonas calceolata*	0.04	1.12	2.66	21					0.22	0.93	1.37	4

Table S4. ANOSIM analysis for surface samples contrasting the effect of season or size-fraction.

Data set	Variable	Statistics	*P*-value
18S filter	season	0.250	0.001
	size fraction	0.376	0.001
16S filter	season	0.216	0.010
	size fraction	0.412	0.001

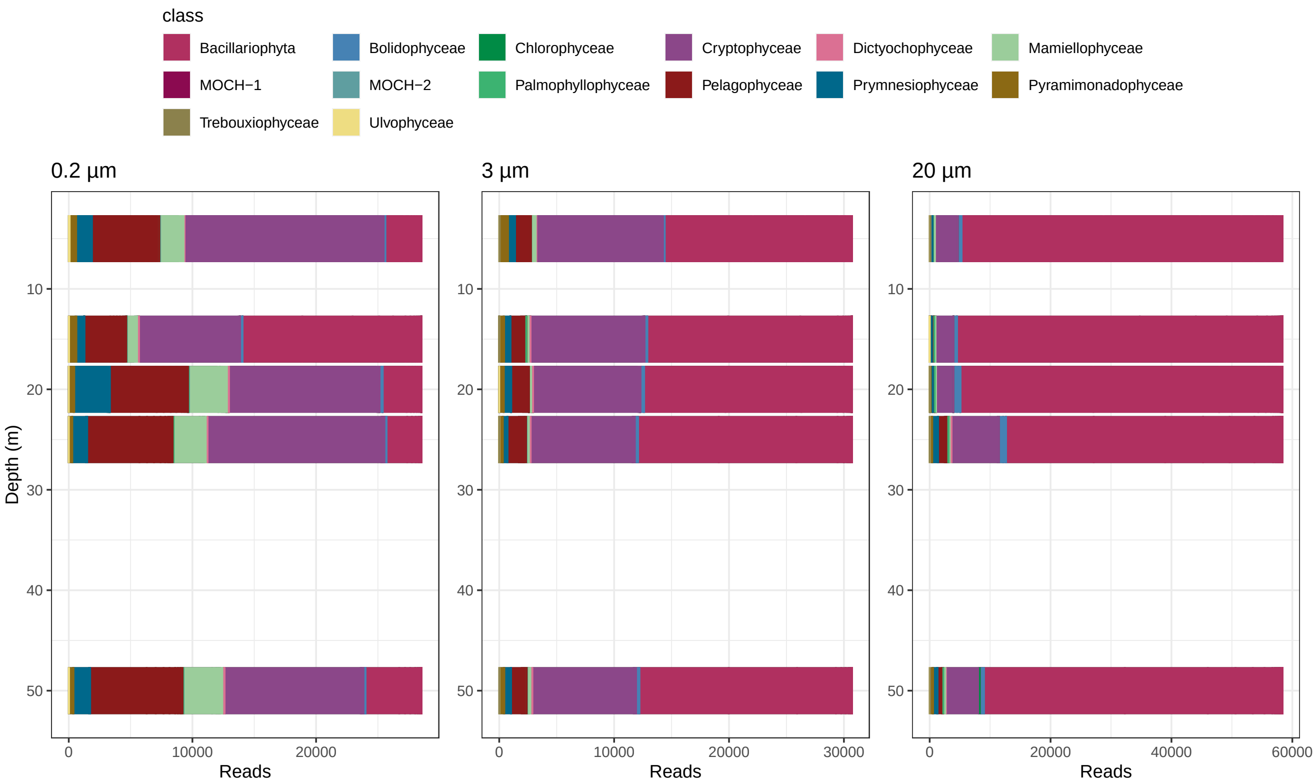

Figure S1. Community composition of phytoplankton at the class level along a vertical profile obtained on January 16, 2015, from 5 m and down to 50 m, based on the 18S rRNA gene for filtered samples.

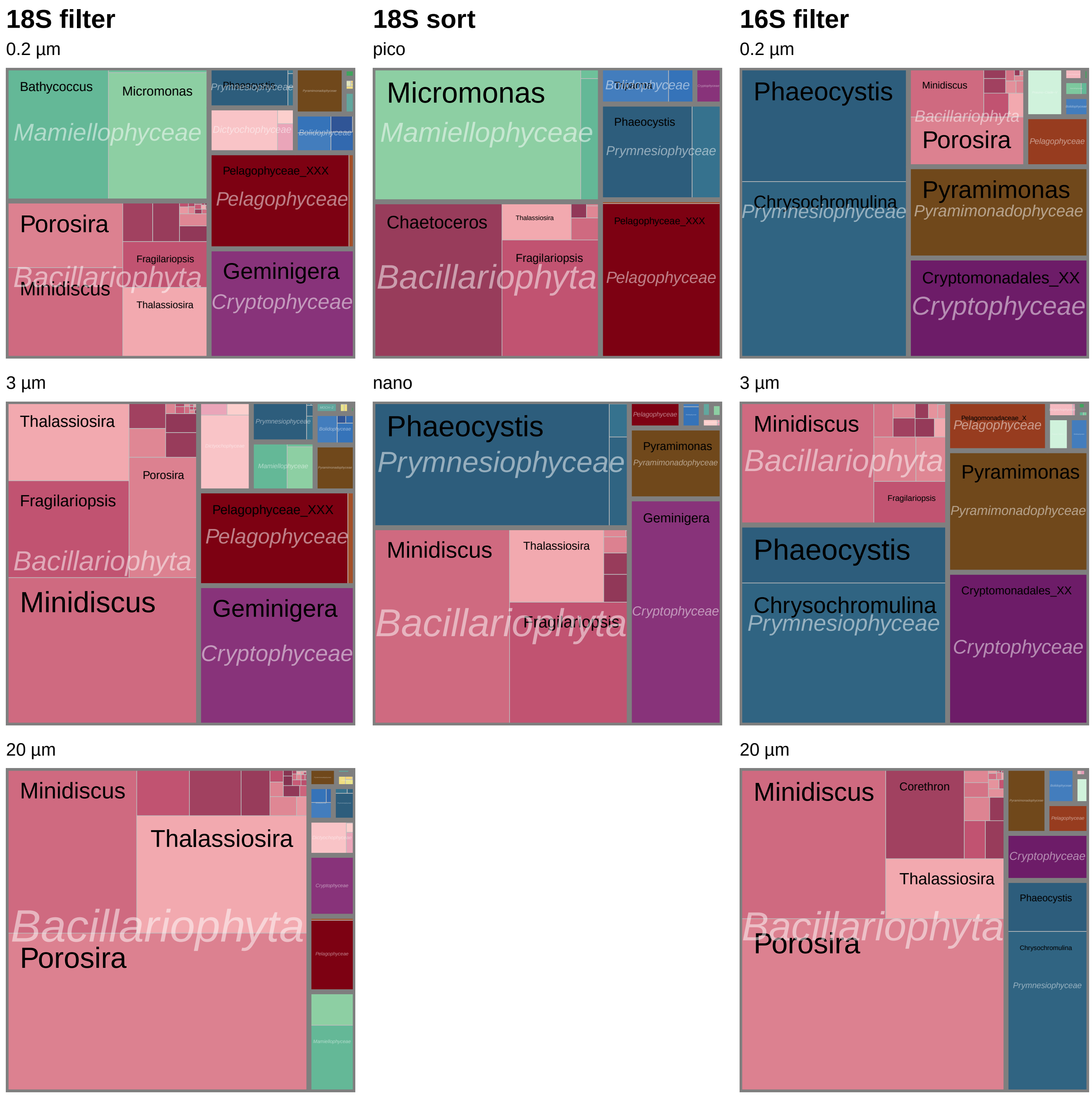

Figure S2. Relative abundance of the different genera in surface samples based on three metabarcoding approaches for each size fraction. Left: 18S rRNA gene on filtered samples. Middle: 18S rRNA gene on sorted samples. Right: plastidial 16S rRNA gene on filtered samples.

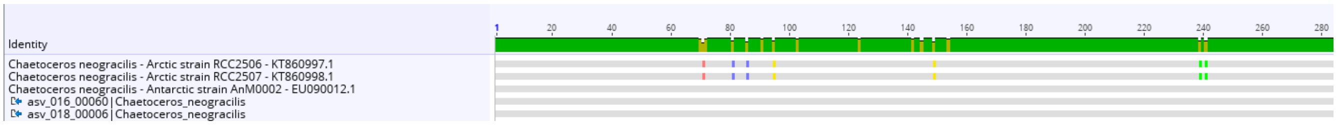

Figure S3. Sequence alignment of 18S rRNA ASVs for *Chaetoceros neogracilis* showing the differences between Arctic and Antarctic strains sequences. The ASVs from this study are identical to the Antarctic strain and show 7 bp differences to Arctic strains.

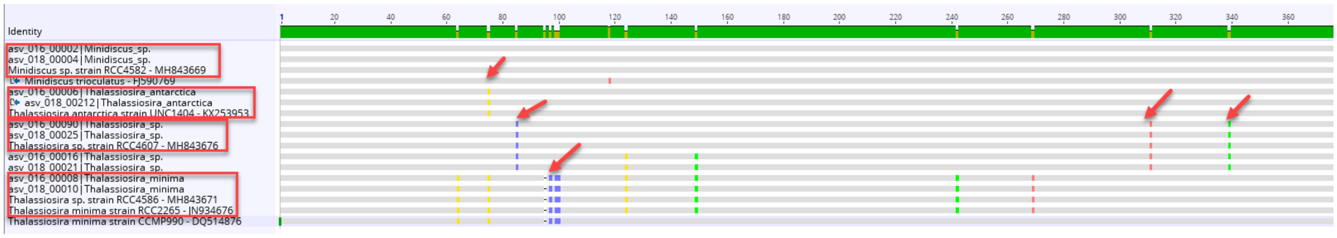

Figure S4. Sequence alignment of 18S rRNA ASVs for major *Thalassiosira* and *Minidiscus* ASVs in comparison to reference sequences.

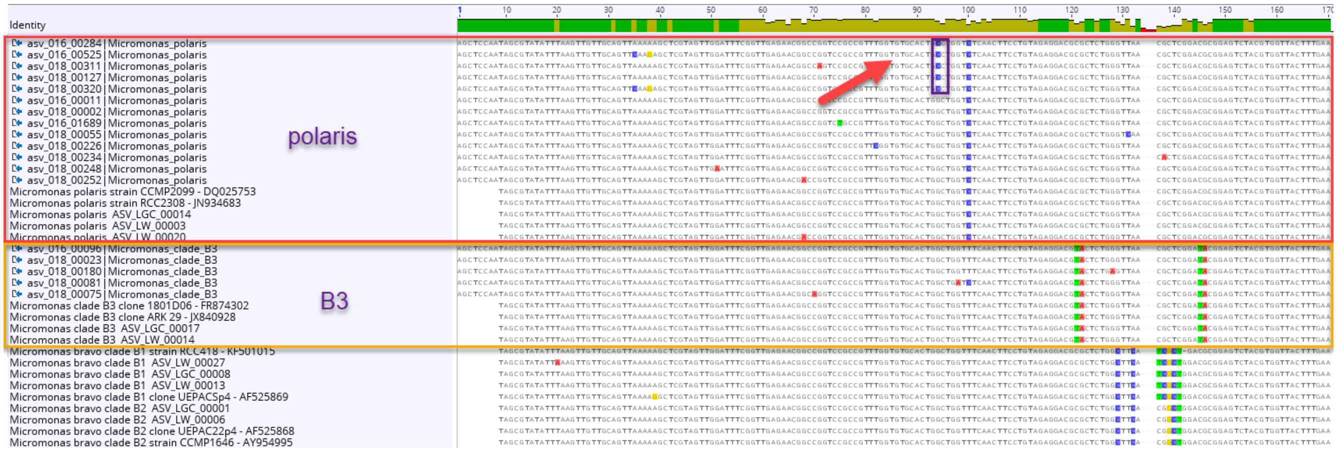

Figure S5. Sequence alignment of 18S rRNA ASVs for *Micromonas* showing the clear signatures for *M. polaris* and clade B3 (Tragin and Vaulot, 2019) Within *M. polaris* some sequences have a different signature pointing to a new clade specific of Antarctic waters (arrow).

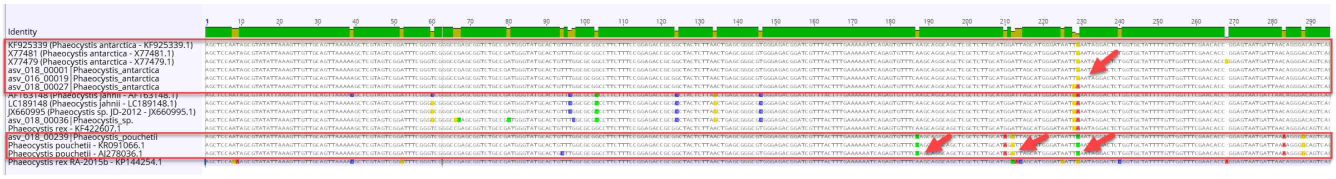

Figure S6. Sequence alignment of 18S rRNA ASVs for *Phaeocystis* showing the clear signatures for *P. antarctica* and *P. pouchetii*.

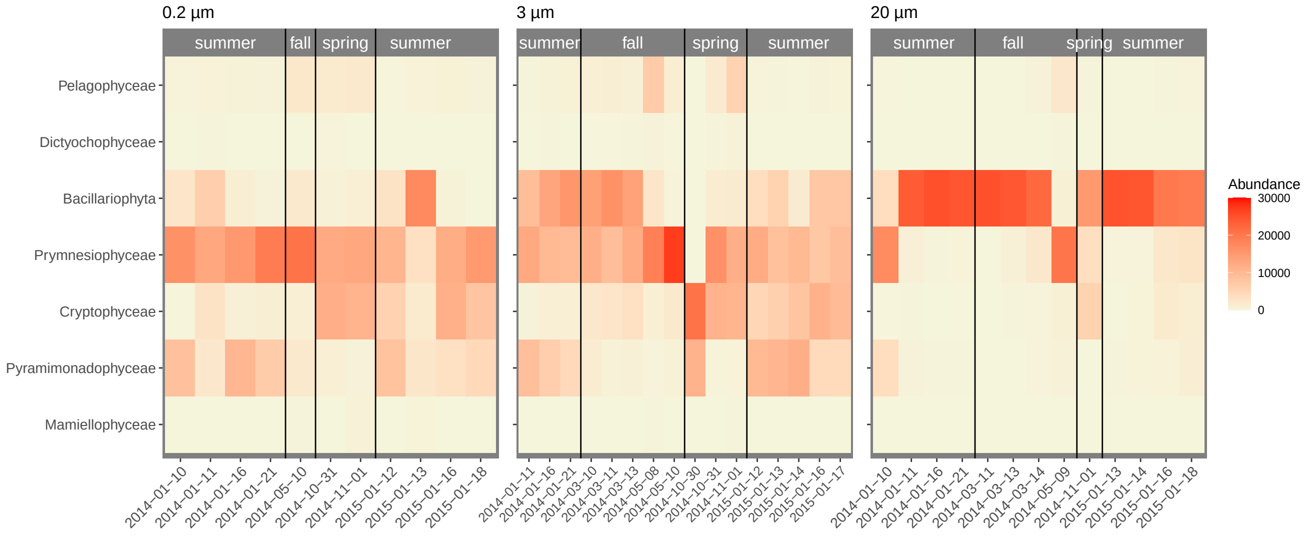

Figure S7. Change in the main phytoplankton groups (excluding dinoflagellates) at class (top) and genus (bottom) levels in Fildes Bay during the study period based on the plastidial 16S rRNA gene in filtered surface samples. The color scale of the heatmap corresponds to the normalized number of reads of each taxon. Season delimitation corresponds to meteorological seasons. Left: 0.2-3 μm. Middle: 3-20 μm. Right: > 20 μm.

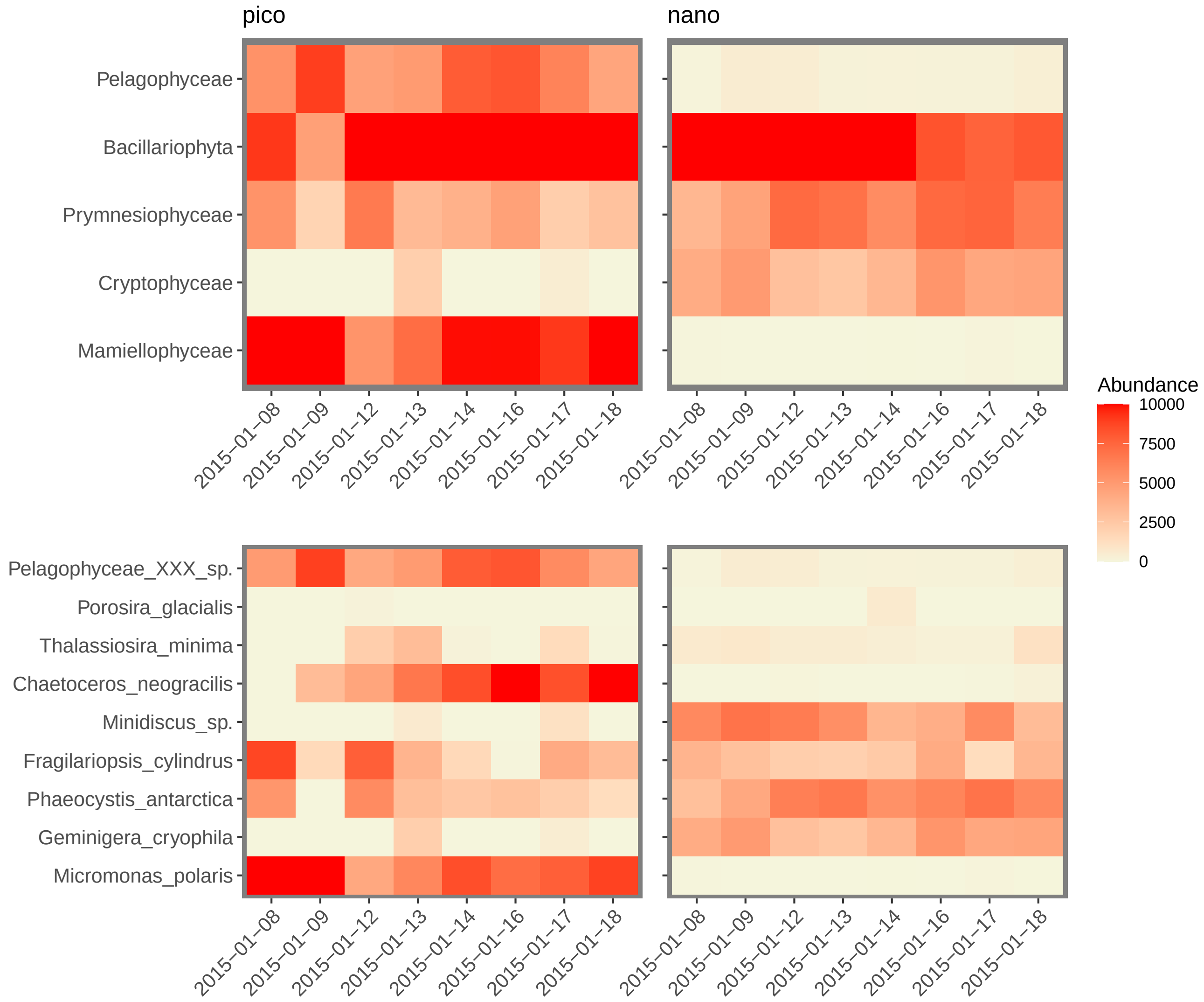

Figure S8. Change in the main phytoplankton groups (excluding dinoflagellates) at class (top) and genus (bottom) levels in Fildes Bay during the summer 2015 based on the 18S rRNA gene for sorted samples from surface waters. The color scale of the heatmap corresponds to the normalized number of reads of each taxon. Left: pico size fraction. Right: nano size fraction.

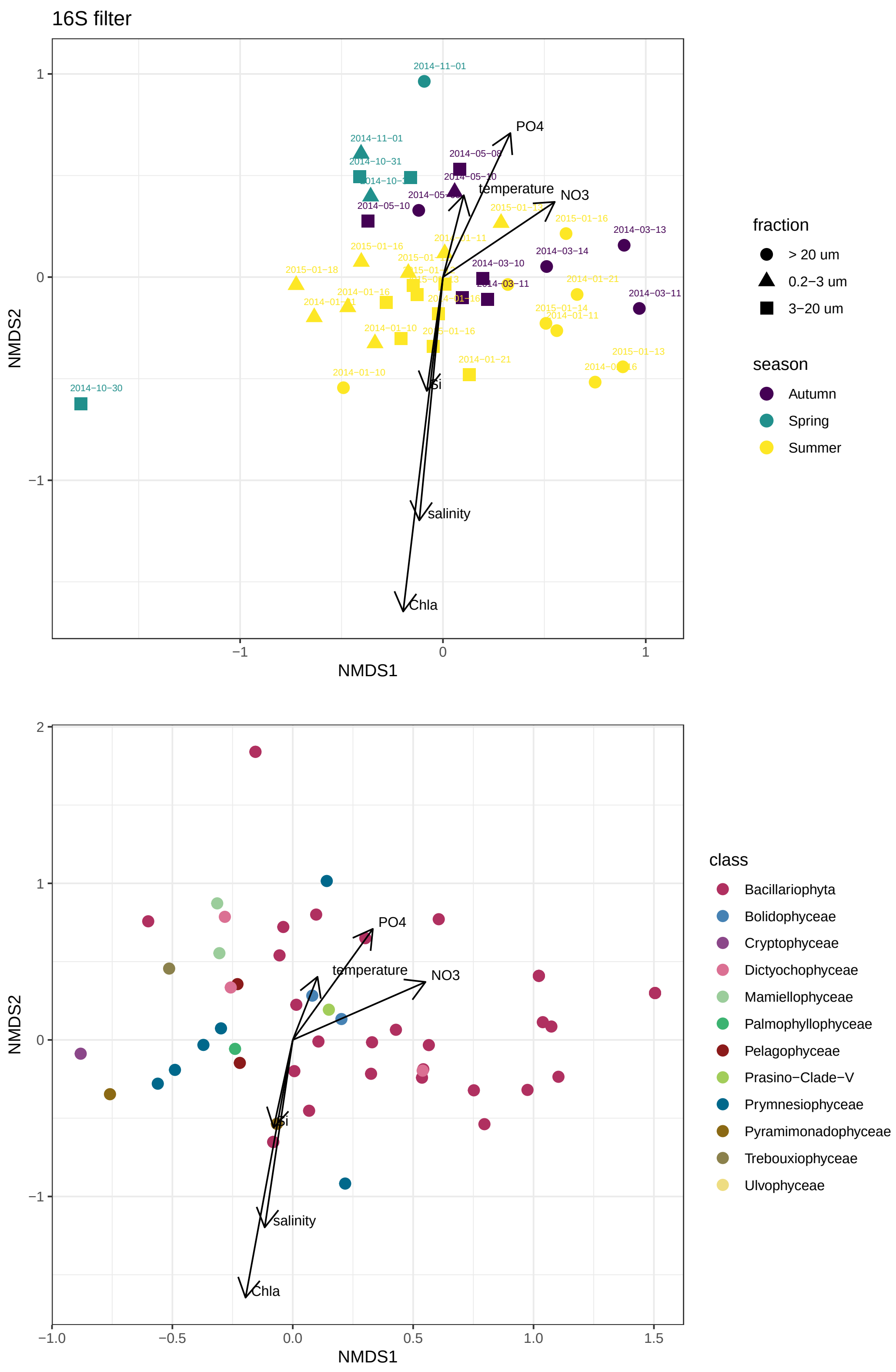

Figure S9. Non-metric multidimensional scaling (NMDS) analysis based on Bray-Curtis dissimilarities of the phytoplankton community composition (species) labeled by meteorological season and size fraction using the plastidial 16S rRNA gene. (A) Samples. (B) ASVs. Stress = 0.15.
